# GLP-1 Receptor Agonists in the Rehabilitation of Patients with Heart Failure: Mechanisms, Clinical Evidence, and Future Perspectives

**DOI:** 10.3390/nu18111688

**Published:** 2026-05-25

**Authors:** Luh Oliva Saraswati Suastika, Yasuko K. Bando, Keiji Hoshino, Norimichi Koitabashi, Yukihiro Saito, Shinsuke Yuasa, Kazufumi Nakamura

**Affiliations:** 1 Department of Cardiovascular Medicine, Okayama University Graduate School of Medicine, Dentistry and Pharmaceutical Sciences, Okayama 700-8558, Japan; oliva.saraswati@gmail.com (L.O.S.S.); p5438a3l@s.okayama-u.ac.jp (Y.S.); yuasa@okayama-u.ac.jp (S.Y.); 2 Center for Advanced Heart Failure, Okayama University Hospital, Okayama 700-8558, Japan; 3 Department of Cardiology and Vascular Medicine, Faculty of Medicine, Udayana University, Denpasar 80361, Indonesia; 4 Department of Molecular Physiology and Cardiovascular Biology, Mie University Graduate School of Medicine, Tsu 514-8507, Japan; ybando@med.mie-u.ac.jp; 5 Division of Cardiology, Gunma Prefectural Cardiovascular Center, Maebashi 371-0004, Japan; k025706@gmail.com (K.H.); norikoitabashi@gmail.com (N.K.); 6 Division of Cardiology, Shimane University Faculty of Medicine, Izumo 693-8501, Japan

**Keywords:** GLP-1 receptor agonists, heart failure, cardiac rehabilitation, obesity, sarcopenia

## Abstract

Heart failure (HF) remains associated with high morbidity and mortality, with heart failure with preserved ejection fraction (HFpEF) becoming increasingly prevalent and therapeutically challenging despite advances in pharmacological and rehabilitative care. Beyond their glucose-lowering effects, glucagon-like peptide-1 receptor agonists (GLP-1RAs) confer cardiometabolic benefits and may serve as effective adjuncts to cardiac rehabilitation (CR), particularly in obese patients with HFpEF. Obesity plays a central role in the pathophysiology of HFpEF, and GLP-1RAs promote weight loss, reduce insulin resistance and leptin signaling, and improve hemodynamic and metabolic abnormalities associated with HFpEF. Accumulating evidence suggests that the benefits of GLP-1RAs are phenotype-specific and more pronounced in patients with HFpEF than in patients with HF with reduced ejection fraction. Current clinical guidelines recommend GLP-1RAs for patients who have type 2 diabetes mellitus and established cardiovascular (CV) disease or are at high CV risk, with recent updates recognizing their potential benefits in patients with HFpEF and obesity. Cardiac rehabilitation, delivered through multidisciplinary programs, remains a cornerstone of HF management. Although caloric restriction and aerobic exercise can be beneficial in patients with HFpEF and obesity, these interventions alone are often insufficient. Sarcopenia is common in older patients with HFpEF and contributes to adverse outcomes, underscoring the importance of incorporating resistance training into CR programs. The most frequent adverse effects of GLP-1RAs are gastrointestinal events, which are generally mild to moderate but may lead to treatment discontinuation in some patients. Future studies should investigate the potential synergistic effects of GLP-1RAs and CR, clarify their long-term safety and efficacy in HF populations, and define their role beyond obese HFpEF phenotypes.

## 1. Introduction

Heart failure (HF) affects more than 64 million people worldwide and remains a leading cause of morbidity and mortality in both developed and developing countries [1,2]. Despite significant advances in pharmacotherapy and device-based interventions, the prognosis of HF remains poor, with mortality rates comparable to or exceeding those of many malignant diseases in certain populations [3]. Among HF phenotypes, the prevalence of heart failure with preserved ejection fraction (HFpEF) is steadily increasing, largely driven by population aging and the growing burden of obesity, hypertension, and diabetes mellitus (DM). HFpEF carries a prognosis similar to that of heart failure with reduced ejection fraction (HFrEF) and presents unique therapeutic challenges due to its complex, multisystem pathophysiology [4,5].

Cardiac rehabilitation (CR) is a cornerstone of contemporary HF management. Evidence from randomized trials and meta-analyses demonstrates that CR improves exercise tolerance, quality of life (QOL), skeletal muscle function, and overall clinical outcomes in patients with HF [6,7]. Nevertheless, participation in CR programs remains suboptimal worldwide, highlighting the need for additional therapeutic strategies that may enhance or complement rehabilitation-based interventions.

Glucagon-like peptide-1 receptor agonists (GLP-1RAs) were initially developed as glucose-lowering therapies for type 2 diabetes mellitus (T2DM). However, accumulating evidence indicates that GLP-1RAs exert physiological effects far beyond glycemic control, including weight reduction, improvements in endothelial function, anti-inflammatory actions, modulation of appetite, and overall enhancements in cardiometabolic health [8]. Large cardiovascular outcome trials (CVOTs), including LEADER, SUSTAIN-6, HARMONY Outcomes, and SURPASS-CVOT, have demonstrated clinically meaningful reductions in major adverse cardiovascular events (MACEs) among high-risk T2DM populations treated with GLP-1RAs [9,10,11,12]. Mechanistically, GLP-1 signaling influences systemic inflammation, vascular homeostasis, and metabolic efficiency—pathophysiological processes closely linked to the development and progression of HF [8,13,14].

More recently, GLP-1RAs have gained considerable attention in the context of HFpEF, particularly among patients with obesity. The STEP-HFpEF trial demonstrated that semaglutide led to substantial improvements in HF-related symptoms, exercise capacity, inflammatory biomarkers, and body weight in obese patients with HFpEF [15]. Similarly, in the SUMMIT trial, treatment with tirzepatide in patients with HFpEF and obesity, with or without T2DM, resulted in a lower risk of a composite endpoint of CV death or worsening heart failure with improved health status compared with placebo [16]. These findings suggest a potential role for GLP-1RAs as adjunctive therapies within rehabilitation-oriented HF management strategies.

This review aims to (1) summarize the mechanistic effects of GLP-1RAs relevant to CV and metabolic systems; (2) critically evaluate the clinical evidence supporting their use in HF; (3) discuss their integration with CR strategies; (4) outline potential risks and safety considerations associated with GLP-1RA therapy; and (5) highlight future research directions, including precision medicine and phenotype-specific therapeutic approaches. The significance and precautions of CR in patients receiving GLP-1RAs are discussed in this article. In particular, nutritional management and maintenance of skeletal muscle mass through CR are considered important.

## 2. Basic Mechanisms and Cardiovascular Effects of GLP-1RAs

### 2.1. Physiological Roles and Pharmacological Profile of GLP-1

1. Discovery and Molecular Biology of GLP-1

GLP-1 is an incretin hormone derived from the proglucagon gene, which is expressed in intestinal L-cells, pancreatic α-cells, and discrete neuronal populations in the brainstem [8]. In intestinal L-cells, tissue-specific post-translational processing by prohormone convertase 1/3 generates biologically active GLP-1 peptides, primarily GLP-1(7–36) amide and GLP-1(7–37). These peptides are secreted rapidly in response to glucose ingestion and exert glucose-dependent insulinotropic effects, suppress glucagon secretion, delay gastric emptying, promote satiety, and reduce appetite.

Endogenous GLP-1 has a very short plasma half-life of approximately 1–2 min due to rapid degradation by dipeptidyl peptidase-4 (DPP-4) and renal clearance. This pharmacokinetic limitation led to the development of GLP-1 receptor agonists (GLP-1RAs), which are structurally modified to resist DPP-4 cleavage and achieve sustained receptor activation.

2. GLP-1 Receptor Structure and Signal Transduction

GLP-1 mediates its biological effects via the GLP-1 receptor (GLP-1R), a G-protein-coupled receptor with seven transmembrane domains. Ligand binding primarily activates the stimulatory G-protein (Gs), leading to increased intracellular cyclic adenosine monophosphate (cAMP) levels and activation of protein kinase A (PKA). In pancreatic β-cells, this signaling cascade enhances intracellular calcium influx and insulin granule exocytosis in a glucose-dependent manner.

Beyond the pancreas, GLP-1R signaling engages additional intracellular pathways, including phosphatidylinositol-3-kinase (PI3K)/Akt and mitogen-activated protein kinase (MAPK), which regulate cell survival, mitochondrial function, and anti-apoptotic responses [1,2]. In the cardiac sinoatrial node, PKA-dependent modulation of calcium cycling proteins accelerates pacemaker activity, providing a mechanistic basis for the modest heart rate increase observed with GLP-1RA therapy [2,3]. GLP-1RAs also act on receptors in the paraventricular nucleus of the hypothalamus, modulating sympathetic nerve activity and contributing to tachycardia [17].

3. Tissue Distribution of GLP-1R

**Figure 1:**
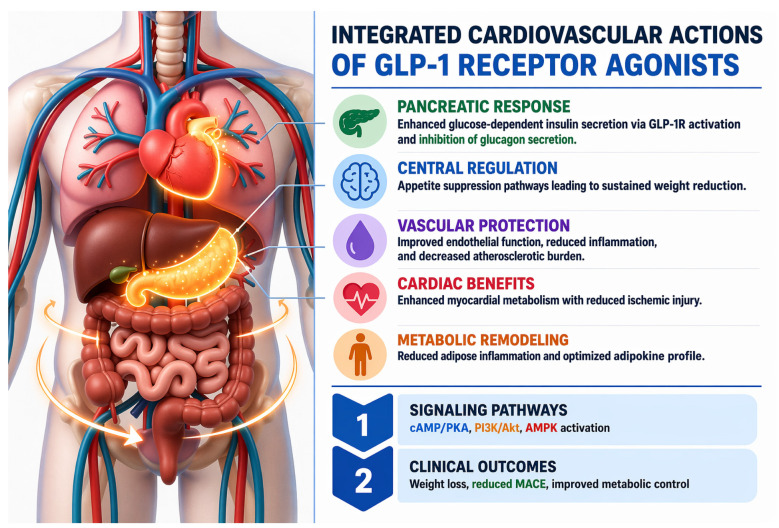
Integrated cardiovascular actions of GLP-1 receptor agonists. The arrows around the abdominal region illustrate the systemic metabolic and gastrointestinal effects of GLP-1 receptor agonists, including modulation of energy balance, adiposity, and gut-related pathways. GLP-1: glucagon-like peptide-1; cAMP: cyclic adenosine monophosphate; PKA: protein kinase A; PI3K: phosphoinositide 3-kinase; Akt: protein kinase B; AMPK: AMP-activated protein kinase; MACE: major adverse cardiovascular events; AMP: adenosine monophosphate; ATP: adenosine triphosphate.

Although GLP-1R expression is most abundant in pancreatic β-cells, it is also detected in the central nervous system, gastrointestinal (GI) tract, kidneys, and CV tissues [2,3]. In the heart and vasculature, GLP-1R is expressed in cardiomyocytes, endothelial cells, vascular smooth muscle cells, and cells of the cardiac conduction system, albeit at lower levels than in endocrine tissues. This widespread receptor distribution underpins the pleiotropic actions of GLP-1RAs and supports the concept of GLP-1 as a multi-organ regulatory hormone (Figure 1).

### 2.2. Beyond Glucose Lowering: Weight Reduction, Inflammation, and Atherosclerosis

1. Body Weight and Energy Balance

Weight reduction is a prominent non-glycemic effect of GLP-1RAs. Central GLP-1R activation in the hypothalamus and brainstem suppresses appetite, enhances satiety, and modulates reward-related feeding behavior, resulting in sustained reductions in caloric intake [8,18]. Although modest increases in energy expenditure via brown adipose tissue activation have been proposed, appetite suppression, delayed gastric emptying, and enhanced satiety remain the primary mechanisms driving weight loss in humans.

2. Anti-Inflammatory and Anti-Oxidative Effects

Chronic low-grade inflammation plays a pivotal role in obesity, T2DM, and atherosclerotic cardiovascular disease (ASCVD). GLP-1RAs reduce systemic and vascular inflammation by suppressing pro-inflammatory cytokines such as tumor necrosis factor-α, interleukin-6, and monocyte chemoattractant protein-1 [19,20]. At the molecular level, GLP-1R activation inhibits nuclear factor-κB signaling and attenuates oxidative stress through reduced reactive oxygen species production and enhanced antioxidant capacity. These effects improve endothelial function independently of glycemic control [20].

3. Anti-Atherosclerotic Actions

Experimental studies consistently demonstrate that GLP-1RAs reduce atherosclerotic plaque burden and enhance plaque stability [19,21]. Mechanistically, GLP-1 signaling attenuates macrophage recruitment, inhibits foam cell formation, and limits necrotic core expansion. Importantly, these vascular benefits are observed in normoglycemic models, indicating direct anti-atherosclerotic effects independent of glucose lowering [21].

### 2.3. Direct Effects on Myocardium and Vasculature

GLP-1R activation in cardiomyocytes enhances myocardial glucose uptake, improves mitochondrial efficiency, and activates PI3K/Akt and AMP-activated protein kinase pathways, promoting cell survival and reducing ischemia–reperfusion injury [22,23]. In endothelial cells, GLP-1 signaling increases nitric oxide (NO) bioavailability via endothelial NO synthase activation, improving vasodilation and reducing arterial stiffness. In vascular smooth muscle cells, GLP-1RAs inhibit proliferation and migration, processes central to neointimal formation and restenosis.

Clinically, GLP-1RAs modestly reduce systolic blood pressure and arterial stiffness, contributing to decreased left ventricular (LV) afterload and improved CV efficiency.

Basic experimental studies suggest direct effects of GLP-1RAs on the myocardium, but it is not possible to distinguish between the direct myocardial effects of GLP-1RAs and their indirect effects mediated by weight loss, improved metabolism, and blood pressure reduction. Interestingly, Hattori et al. reported that the expressions of GLP-1R were enhanced in the hearts of patients with HFrEF [24]. Currently, the direct effects are not supported by evidence in humans, and further research is needed to clarify this point.

## 3. GLP-1RAs in HF: Clinical Evidence

### 3.1. Effects in HFpEF and HFrEF

**Figure 2:**
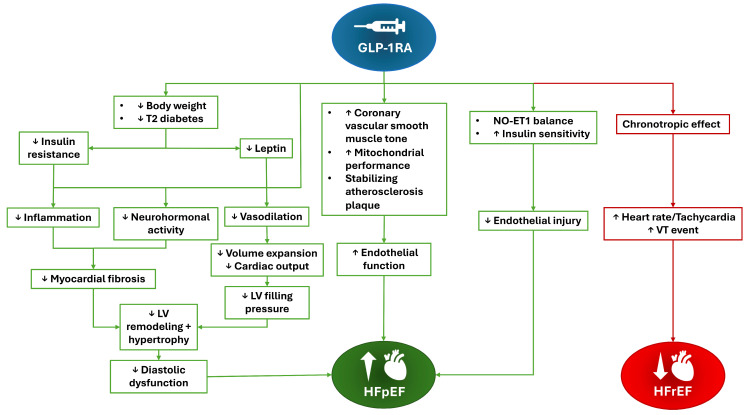
Potential mechanisms of GLP-1 receptor agonists in HFpEF and HFrEF. Green boxes and lines represent mechanisms in HFpEF. Red boxes and lines denote mechanisms in HFpEF. The symbol ↑ inside the box indicates an increase or activation, whereas the symbol ↓ inside the box indicates a decrease or inhibition of the corresponding pathway or process. Connecting arrows between boxes represent the possible directional mechanistic relationships. GLP-1RA: glucagon-like peptide-1 receptor agonist; T2: type 2; NO: nitric oxide; ET1: endothelin-1; VT: ventricular tachycardia; HFpEF: heart failure with preserved ejection fraction; HFrEF: heart failure with reduced ejection fraction.

Over the past two decades, numerous clinical trials have evaluated the CV outcomes associated with GLP-1RAs in patients with T2DM. Hospitalization for HF has typically been included as a secondary endpoint in these CVOTs [18]. A meta-analysis encompassing more than 60,000 T2DM participants demonstrated that GLP-1RA therapy was associated with an 11% reduction in the risk of HF hospitalization [25]. More recently, research has focused on the potential benefits of GLP-1RAs in patients with established HF, with particularly encouraging results in those with HFpEF. Heart failure with preserved ejection fraction represents a complex and heterogeneous syndrome with multiple underlying etiologies. Common comorbidities, including obesity, DM, hypertension, and metabolic syndrome, contribute to its pathophysiology through systemic inflammation, leading to alterations in myocardial structure, impaired cardiomyocyte contractility, and coronary microvascular dysfunction. The therapeutic effects of GLP-1RAs in HFpEF likely derive from several mechanisms (Figure 2), including weight loss and hemodynamic unloading, improved insulin sensitivity, attenuation of inflammation, amelioration of microvascular dysfunction and metabolic state, and modest natriuretic and diuretic effects [26]. While these agents appear to confer indirect cardiac benefits, direct myocardial effects of GLP-1RAs remain uncertain.

Obesity is strongly associated with HFpEF, with up to 80% of HFpEF patients in Western populations being overweight or obese [27,28]. In contrast, in Asia, the prevalence of overweight or obesity among HFpEF patients is lower, approximately 26% overall, but varies between 6% and 38% across East Asian cohorts [29,30,31,32]. In obesity, increased metabolic demands lead to systemic vasodilation, which triggers neurohormonal activation and sodium retention [33]. Leptin secretion from perivascular adipose tissue further contributes to vasodilation [34], resulting in volume expansion, increased cardiac output, and LV remodeling with hypertrophy due to wall stress. The resulting volume overload in obese patients exacerbates elevated LV filling pressures, which are commonly observed due to hypertension or arterial stiffness in approximately 75% of obese patients [35]. Concentric LV remodeling, often associated with insulin resistance, hyperleptinemia, and diabetes, is common in obesity and contributes to myocardial fibrosis in both left and right ventricles [31,35]. Leptin-driven inflammation and neurohormonal activation further promote interstitial fibrosis in obesity [36,37]. Myocardial fibrosis, particularly interstitial fibrosis, contributes to myocardial stiffening and reduced compliance, leading to LV diastolic dysfunction, a hallmark feature of HFpEF [38].

The weight-reducing efficacy of GLP-1RAs is well established and clinically relevant in HFpEF. Weight loss in this population lowers blood pressure, enhances exercise capacity, improves QOL, and reduces New York Heart Association functional class [39]. In obese HFpEF patients, once-weekly semaglutide (2.4 mg) and tirzepatide have demonstrated significant improvements in symptoms, exercise tolerance, and functional status [15,16]. Combined therapy with GLP-1RAs and sodium glucose transporters-2 (SGLT2) inhibitors in T2DM patients with overweight or obesity and HFpEF further reduced HF hospitalizations, new-onset atrial fibrillation, inflammatory biomarkers (e.g., C-reactive protein), acute kidney injury, and the need for renal replacement therapy compared to SGLT2 inhibition alone [40]. Liraglutide has been shown to decrease early LV diastolic filling and LV filling pressures, thus reducing LV overload and improving diastolic function [41]. In a murine HFpEF model, liraglutide improved cardiac function and metabolic parameters, attenuated LV hypertrophy and myocardial fibrosis, and reduced atrial weight, natriuretic peptide levels, and pulmonary congestion [42].

Endothelial dysfunction, a hallmark of HFpEF pathogenesis, affects coronary arteries, myocardial capillaries, and the endocardium [43]. Hypertension and diabetes, the most common HFpEF risk factors, are associated with endothelial impairment in the endocardium and myocardium capillaries [44]. This endothelial dysfunction is related to increased systemic arterial stiffening, contributing to the development of hypertension, which subsequently drives LV remodeling, diastolic dysfunction, and HFpEF progression [45]. Moreover, endothelial dysfunction independently predicts CV outcomes in HFpEF [46]. Glucagon-like peptide-1 receptor agonists have shown benefits in improving endothelial function by enhancing coronary vascular smooth muscle tone, optimizing mitochondrial performance, stabilizing atherosclerotic plaques, and slowing plaque progression [44,47]. They also restore the NO–endothelin-1 (ET-1) balance and improve insulin sensitivity, further mitigating endothelial injury [48].

In a recent large-scale study of more than 8000 patients with HF and mildly reduced or preserved ejection fraction—with or without T2DM and obesity—treatment with GLP-1RAs (exenatide, semaglutide, or tirzepatide) significantly reduced the composite endpoint of CV death or worsening HF as well as worsening HF alone [49]. A meta-analysis by Siddiqui et al. [50] corroborated these findings, showing reductions in LV mass, left atrial volume, and *N*-terminal pro-brain natriuretic peptide (NT pro-BNP) levels among HFpEF patients treated with GLP-1RAs. These findings highlight the beneficial effect of GLP-1RAs in HFpEF management.

Conversely, the evidence supporting GLP-1RA use in HFrEF remains limited and mixed. Smaller clinical studies have raised safety concerns. In HFrEF patients with recent hospitalization, liraglutide did not improve clinical stability (mortality, HF hospitalization, changes in NT-proBNP level, 6 min walking distance, and QOL) compared with placebo [51]. Unlike in HFpEF, GLP-1RAs did not improve left ventricular ejection fraction (LVEF), LV volumes, or global longitudinal strain in HFrEF [50]. The LIVE trial [52] similarly showed no improvement in LV systolic function in stable chronic HFrEF patients with and without DM and noted an average increase of up to six beats per minute in heart rate, along with episodes of ventricular tachycardia (VT), hemodynamically unstable atrial fibrillation, death due to VT, acute coronary syndromes, and worsening HF. Comparable findings were reported by Marques et al. [53]: GLP-1RAs were associated with significant increases in heart rate, number of non-sustained VT events, and total shock/anti-tachycardia pacing. Evidence from a meta-analysis by Greco et al. confirms the increase in HR after chronic GLP-1RA treatment in patients with DM; this HR change is not a consequence of sympathetic or parasympathetic stimulation [54]. Experimental data from Ban et al. and Pyke et al. identified GLP-1R expression predominantly in the atria, especially the sinoatrial node, potentially explaining the chronotropic effects of GLP-1RAs in HFrEF [55,56].

The neutral or potentially adverse outcomes of GLP-1RAs in HFrEF may be related to several mechanisms. Their heart rate-elevating effect is concerning, as higher resting heart rates are associated with a poorer prognosis in HFrEF (Figure 2). In advanced HF patients with cachexia, GLP-1RA-induced weight loss may exacerbate muscle wasting and nutritional decline, potentially inducing clinical deterioration [57]. Furthermore, unlike in HFpEF, GLP-1RAs do not significantly lower NT-proBNP levels in patients with HFrEF [58]. Nevertheless, findings from the large PRAISE-HF observational study of over 1700 patients with HFrEF, T2DM, and obesity suggest that GLP-1RAs, when used at diabetic management doses, appear to be safe, particularly in patients with a body mass index (BMI) > 40 kg/m^2^ [59].

### 3.2. Review of Major Clinical Trials

The CV benefits of GLP-1RAs have been consistently demonstrated in several recent CVOTs. One of the earliest landmark studies was the LEADER trial (Liraglutide Effect and Action in Diabetes: Evaluation of Cardiovascular Outcome Results) published in 2016 [10]. In this trial, more than 9300 patients with T2DM and established CV disease or at high CV risk were followed for a median of 3.8 years. Treatment with liraglutide (up to 1.8 mg daily or the highest tolerated dose) significantly reduced the primary composite endpoint—CV death, nonfatal MI, or nonfatal stroke—compared with placebo (hazard ratio [HR] = 0.87; 95% confidence interval [CI] 0.78–0.97; *p* = 0.01 for superiority). Moreover, liraglutide therapy was associated with reductions in both CV mortality and all-cause mortality. Gastrointestinal adverse events were the most frequent cause of treatment discontinuation. Liraglutide improved glycemic control, as reflected by greater reductions in HbA1c and a lower incidence of hypoglycemia relative to placebo [60].

The SUSTAIN-6 trial (Trial to Evaluate Cardiovascular and Other Long-term Outcomes with Semaglutide in Subjects with Type 2 Diabetes) enrolled 3297 patients with T2DM at high CV risk. Treatment with once-weekly subcutaneous semaglutide (0.5 or 1.0 mg) resulted in a 26% reduction in the primary composite outcome of MACEs compared with placebo (HR 0.74; 95% CI 0.58–0.95), primarily driven by fewer nonfatal stroke events [9]. Similarly, the PIONEER 6 trial (Peptide Innovation for Early Diabetes Treatment) demonstrated that oral semaglutide was non-inferior to placebo for CV risk in more than 3000 T2DM patients with high CV risk [61]. More recently, the SELECT trial (Semaglutide Effects on Cardiovascular Outcomes in People with Overweight or Obesity) showed that weekly injection of semaglutide at 2.4 mg, compared to placebo, significantly reduced MACEs in patients with overweight or obesity without diabetes but with established cardiovascular disease (CVD) [62]. Additional GLP-1RA agents have shown similar benefits. In the Harmony Outcomes trial, once-weekly albiglutide reduced the risk of MACEs when added to standard care in T2DM patients with CVD [11]. The REWIND trial found that dulaglutide injection reduced MACE incidence compared with the placebo group (HR 0.88; 95% CI 0.79–0.99; *p* = 0.026) [63]. The AMPLITUDE-O trial also demonstrated a lower risk of CV events among T2DM patients receiving a weekly subcutaneous injection of efpeglenatide [64]. The CV benefits of tirzepatide are evident in the SURPASS CVOT 2025 and the post hoc analysis of SURMOUNT-5 trials. In the former study, tirzepatide showed non-inferiority in MACE reduction compared to dulaglutide in patients with T2DM and CVD (HR 0.92; 95% CI 0.83–1.01, *p* = 0.003 for non-inferiority [12]. In the SURMOUNT-5 trial, a post hoc analysis demonstrated that tirzepatide was associated with more favorable CV outcomes compared with semaglutide in adults with overweight or obesity [65].

However, not all CVOTs have reported significant benefits. Some CVOTs reported neutral effects of GLP-1RAs on MACEs. In the ELIXA trial (Evaluation of Lixisenatide in Acute Coronary Syndrome), the addition of lixisenatide to standard care in patients with T2DM and recent acute coronary syndrome did not significantly reduce MACEs or other major adverse events (HR 1.02; 95% CI 0.89–1.17) [66]. Similarly, the EXSCEL (Exenatide Study of Cardiovascular Event Lowering) and FREEDOM-1 trials found no significant differences in MACE incidence between exenatide and placebo among T2DM patients with or without pre-existing CVD [67,68].

Beyond diabetes, emerging evidence supports the role of GLP-1RAs in HF, particularly HFpEF. The STEP-HFpEF trial (Semaglutide Treatment Effect in People with Obesity and HFpEF) enrolled 529 patients with HFpEF and obesity and demonstrated that, over a 52-week follow-up, semaglutide at a dose of 2.4 mg significantly improved symptoms, exercise tolerance, and inflammatory markers while reducing body weight across all LVEF subgroups (45–49%, 50–59%, and ≥60%) [15,69]. Similar results were observed in the STEP-HFpEF DM trial, which confirmed these benefits in patients with HFpEF and T2DM [70]. Consistent findings were also reported in a retrospective analysis from the Swedish Heart Failure Registry (SwedeHF), which included heart failure patients with a BMI of >25 kg/m^2^. GLP-1RA treatment was associated with reduced CV mortality, particularly in those with a BMI ≥ 30 kg/m^2^ and in patients with LVEF < 40% compared with those with LVEF > 40% [71]. The SUMMIT trial also demonstrated that, in patients with HFpEF (LVEF of at least 50%) and obesity (BMI > 30 kg/m^2^), tirzepatide reduced the risk of a composite endpoint of CV death or worsening HF compared to placebo (HR 0.62; 95% CI 0.41–0.95; *p* = 0.026). These benefits of tirzepatide were consistent regardless of the presence or absence of type 2 diabetes. Tirzepatide also significantly improved health status, as assessed by the mean change in the Kansas City Cardiomyopathy Questionnaire (KCCQ)-Clinical Summary Score (between-group difference 6.9, 95% CI 3.3–10.6, *p* < 0.01) [16]. It should be noted that the definition of HFpEF varied among the clinical studies cited in this paper. The STEP-HFpEF trial [15] and STEP-HFpEF DM trial [70] included patients with LVEF ≥ 45%, and the SUMMIT trial [16] included patients with LVEF ≥ 50%. The SwedeHF registry [71] included patients treated with GLP-1RA; 20% had an LVEF ≥ 50%, 23% had an LVEF of 40–49%, and 57% had an LVEF < 40.

It is unclear to what extent benefits attributed to HFpEF might reflect treatment of obesity rather than HF pathophysiology itself. Since the beneficial effects of GLP-1RAs were observed along with weight changes in the STEP-HFpEF, STEP-HFpEF DM, and SUMMIT trials, it is now recommended to interpret GLP-1RA therapy in a phenotype-specific manner for HFpEF patients, rather than considering the general therapeutic effect for all HFpEF patients.

In contrast, earlier smaller trials in HFrEF raised safety concerns. The FIGHT (Functional Impact of GLP-1RA for Heart Failure Treatment) and LIVE (Effect of Liraglutide on Left Ventricular Function in Stable Chronic Heart Failure) trials reported that liraglutide did not reduce mortality or hospitalization rates or improve LV systolic function. Moreover, it may pose potential risks in advanced HFrEF [51,52]. However, the large observational PRAISE-HFrEF study found no significant difference in the time to first HF hospitalization or all-cause mortality between HFrEF patients treated with GLP-1RAs and those receiving DPP-4 inhibitors or sulfonylureas (HR 0.97; 95% CI 0.85–1.23; *p* = 0.74). This suggests that standard-dose GLP-1RA therapy may be safe in select patients with HFrEF and obesity, particularly those with a BMI ≥ 40 kg/m^2^ [59]. In recent large multicenter real-world cohorts of patients with HFrEF and overweight or obesity, the addition of GLP-1RAs to quadruple GDMT was associated with lower mortality [72]. Collectively, these findings indicate that GLP-1RAs confer robust CV benefits in patients with HFpEF, particularly with overweight and obesity, while appearing safe in appropriately selected patients with HFrEF.

Mechanistic considerations may partly explain the heterogeneous effects of GLP-1RAs in HFrEF. Unlike HFpEF, which is strongly associated with obesity-related systemic inflammation and metabolic dysfunction, HFrEF is often characterized by primary myocardial injury and neurohormonal activation, conditions in which the indirect metabolic benefits of GLP-1RAs may be less pronounced. Furthermore, the modest increases in heart rate observed with GLP-1RA therapy have raised concerns regarding potential adverse effects in patients with advanced systolic dysfunction. However, these signals were primarily derived from small-scale trials, and subsequent real-world studies have not consistently demonstrated excess risk. Importantly, emerging evidence suggests that selected subgroups of patients with HFrEF—particularly those with obesity, insulin resistance, or type 2 diabetes mellitus—may still derive metabolic and prognostic benefits from GLP-1RA therapy when used alongside guideline-directed medical therapy. Taken together, current evidence indicates that GLP-1RAs should not be considered routine therapy for HFrEF at present, but they may represent a reasonable adjunctive option in carefully selected patients, especially those with cardiometabolic comorbidities.

### 3.3. Position in International and Domestic Guidelines

Multiple international and regional guidelines and consensus statements have incorporated evidence from CVOTs to guide the use of GLP-1RAs in T2DM (Table 1). In patients with T2DM and either established ASCVD or ASCVD risk factors, GLP-1RAs have demonstrated efficacy comparable to that of SGLT2 inhibitors in reducing MACEs, while producing greater improvements in glycemic parameters such as HbA1c and fasting plasma glucose. However, most CVOTs were not specifically designed to evaluate HF outcomes. Although recent clinical trials have demonstrated the benefits of GLP-1RA therapy in patients with HFpEF and obesity, current guidelines do not yet provide specific recommendations for their use in this population.

The American Diabetes Association Standards of Care in Diabetes 2025 [73] recommend that in patients with T2DM and either established CVD or multiple ASCVD risk factors, a GLP-1RA—either alone or in combination with an SGLT2 inhibitor—should be used to reduce the risk of MACEs (level of evidence A). Furthermore, for patients with T2DM, obesity, and HFpEF, the ADA specifically endorses GLP-1RAs with proven benefits for improving HF symptoms, exercise capacity, and physical function, also supported by level of evidence A. Similarly, the 2023 European Society of Cardiology guidelines for CVD management in patients with diabetes recommend the use of GLP-1RAs with established CV benefit in individuals with T2DM and ASCVD to reduce CV events—independent of baseline or target HbA1c levels and irrespective of concomitant glucose-lowering medications (Class I recommendation, level of evidence A). Given their generally neutral effects on the risk of HF hospitalization, GLP-1RAs such as lixisenatide, liraglutide, semaglutide, exenatide, dulaglutide, and efpeglenatide are also suggested as glucose-lowering options for patients with T2DM who have or are at risk for HF [74].

The Japanese Circulation Society provides more specific recommendations. Semaglutide may be considered for patients with HF and LVEF of >45% and obesity (BMI > 30 kg/m^2^) to reduce CV death and HF hospitalization (Class IIa recommendation). Tirzepatide is also recommended for consideration in patients with HF (LVEF > 50%) and obesity to reduce similar outcomes [75]. Conversely, the Canadian Cardiovascular Society Heart Failure Guidelines [76] currently do not issue specific recommendations regarding GLP-1RA use in HF. Although GLP-1RAs have demonstrated efficacy in reducing ASCVD events among patients with T2DM, their role in preventing HF onset and their long-term safety in established HF patients remain areas of ongoing investigation.

Clinicians should recognize that guideline endorsement of GLP-1RAs is currently recommended for patients with T2DM and established or high-risk ASCVD. On the other hand, evidence in HFpEF is driven primarily by trials in obesity-related HFpEF.

## 4. Integration with Cardiac Rehabilitation

### 4.1. Objectives and Structure of Cardiac Rehabilitation

The principal aims of CR in HF are to improve exercise tolerance and health-related QOL, preserve muscle strength and mass to enhance functional independence, and ultimately improve clinical prognosis [6,77]. Peak oxygen uptake (peak VO_2_), a key indicator of exercise capacity, is closely associated with prognosis and QOL in patients with HF; its improvement is therefore a primary purpose of CR [78,79]. Regarding QOL, symptom burden and functional status—typically assessed using instruments such as the KCCQ—are prioritized outcomes [80]. CR integrates comprehensive, multidisciplinary strategies to accomplish these goals. In patients with HFpEF, as in HFrEF, the efficacy of CR—centered on appropriately prescribed exercise training—has been demonstrated, and CR should be offered to all patients who are able to exercise [81,82].

**Figure 3:**
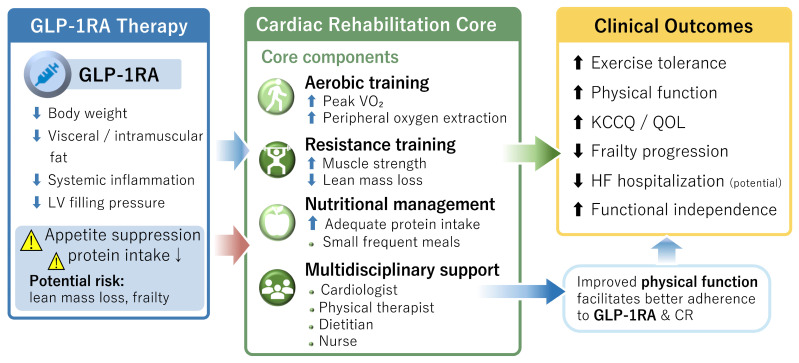
An integrated cardiac rehabilitation (CR) strategy for patients with obesity-related HFpEF treated with GLP-1 receptor agonists (GLP-1RAs). Blue arrows indicate synergistic integration between GLP-1RA therapy and CR, whereby pharmacologically induced weight loss is translated into functional benefits. Red arrows denote the mitigation of potential GLP-1RA–associated adverse effects, including lean mass loss and frailty, through resistance training, nutritional management, and multidisciplinary care within CR. CR, consisting of aerobic exercise, resistance training, nutritional management, and multidisciplinary support, plays a central role in improving exercise tolerance and quality of life in obesity-related HFpEF. Upward arrows indicate improvement or increase, whereas downward arrows indicate reduction or attenuation. CR: cardiac rehabilitation; GLP-1RA: glucagon-like peptide-1 receptor agonist; HF: heart failure; HFpEF: heart failure with preserved ejection fraction; KCCQ: Kansas City Cardiomyopathy Questionnaire; LV: left ventricular; QOL: quality of life; VO_2_: oxygen uptake.

The structure of CR programs is based on coordinated, multidisciplinary care. Exercise training constitutes the core component, targeting improvements in exercise tolerance using both aerobic training to enhance cardiorespiratory fitness and resistance training to increase muscular strength [77]. In HFpEF, training effects manifest predominantly as peripheral adaptations—improvements in skeletal muscle function and widening of the arteriovenous oxygen difference—accompanied by increases in peak VO_2_ and gains in QOL [83]. In addition, patient education and lifestyle counseling are provided to strengthen self-management skills, support adherence to pharmacotherapy, and address modifiable risk factors (e.g., smoking cessation, sodium restriction, and weight management). Nutritional counseling and psychosocial support are essential elements delivered through a multidisciplinary team involving physicians, nurses, physical therapists, and dietitians (Figure 3) [77,84]. Over the long term, these interventions may lower the risk of rehospitalization for heart-failure decompensation and favorably influence long-term prognosis [7,79].

### 4.2. Obesity-Related HFpEF and the Role of Body Composition

Obesity is highly prevalent in HFpEF, with reports from the United States and Western Europe indicating that approximately 80% of patients are overweight or obese [85]. Obesity is independently associated with the development and progression of HFpEF and, through coexisting systemic inflammation, hypertension, insulin resistance, and dyslipidemia, adversely affects not only the CV system but also skeletal muscle function, thereby reducing exercise tolerance [34,86]. Increased visceral, epicardial, and intermuscular adipose tissues correlate with impaired skeletal muscle function and reduced peak VO_2_ [87]. Advanced imaging studies, including those by Haykowsky et al., demonstrate that regional fat distribution predicts exercise intolerance more strongly than total adiposity, underscoring the importance of qualitative as well as quantitative abnormalities in adipose tissue [88].

In a pivotal randomized trial, caloric restriction and aerobic exercise each improved peak VO_2_ in older patients with obese HFpEF, with the combination producing additive gains [27]. However, inter-individual variability in weight loss and symptom improvement remains considerable, and lifestyle modifications alone are insufficient for many patients [27]. These observations have prompted increasing interest in adjunctive pharmacologic approaches to address obesity-related HFpEF.

### 4.3. GLP-1 Receptor Agonists in HFpEF with Obesity

The STEP-HFpEF and STEP-HFpEF DM trials demonstrated that once-weekly semaglutide yields clinically meaningful improvements in symptoms, physical function, body weight, and inflammatory biomarkers in patients with obesity-related HFpEF [15,70]. A subsequent pooled analysis confirmed consistent benefits across diverse patient populations, reinforcing the robustness of GLP-1RAs as therapeutic options in this phenotype [89].

Emerging evidence from next-generation incretin-based therapies further expands this potential. Tirzepatide, a dual GIP/GLP-1RA, has shown substantial weight reduction and improvement in health status in patients with HFpEF with obesity, suggesting that incretin-based pharmacotherapy may continue to evolve as a cornerstone in cardiometabolic HF management [16]. Reflecting these developments, the 2025 American College of Cardiology Scientific Statement highlights GLP-1-based therapies as promising adjuncts for managing obesity and related cardiometabolic abnormalities in HF. Recent reviews also emphasize their multifaceted effects on systemic inflammation, metabolic regulation, and physical function [90].

### 4.4. Strategies to Preserve Muscle Mass and Prevent Frailty

Lean mass abnormalities, including sarcopenia, sarcopenic obesity, and muscle fat infiltration, are increasingly recognized as central contributors to impaired physical function and poor prognosis in HF. These abnormalities appear to be particularly prominent in older adults with HFpEF, underscoring the need for rehabilitation strategies that explicitly target muscle preservation [91]. Many patients with HFpEF are older adults, a population in whom sarcopenia is highly prevalent. Previous studies have shown that HFpEF is characterized not only by advanced age but also by substantial deficits in skeletal muscle mass, strength, and quality, including increased intramuscular fat [87]. Frailty contributes to functional decline in activities of daily living and increases fall risk, and it is associated with adverse outcomes in HF [92]. Because declines in muscle strength and mass lie at the core of frailty [93] and skeletal muscle impairments play a central role in exercise intolerance in HFpEF [87], CR must implement systematic strategies to preserve muscle mass. Indeed, in interventional trials targeting older adults with obesity and HFpEF, the magnitude of improvement in peak VO_2_ correlates significantly with gains in lean body mass (skeletal muscle) and with reductions in thigh muscle fat infiltration [94,95]. Accordingly, CR should incorporate not only aerobic training to enhance endurance but also resistance training and related modalities to strengthen muscle and maintain muscle mass. For frailty accompanying HFpEF, multicomponent training that addresses strength, balance, mobility, and endurance is beneficial for improving physical function [96], and comprehensive programs tailored to individual capabilities are recommended [97].

### 4.5. Nutritional Management and Multidisciplinary Integrated Support

Although weight loss with GLP-1RAs is primarily attributable to reductions in fat mass, decreases in lean mass have also been observed. In dual-energy X-ray absorptiometry analyses, lean mass accounted for approximately 35% of total weight loss with semaglutide in STEP 1 [98] and about 25% with tirzepatide in SURMOUNT-1 [99]. Although the clinical relevance of these changes remains uncertain, particularly in older adults, a reduction in protein intake or insufficient physical activity could potentially exacerbate age-related declines in muscle quantity and quality [100,101]. Importantly, a recent analysis cautioned that weight-loss pharmacotherapy in HFpEF—particularly in older adults—may exacerbate pre-existing frailty or sarcopenia if nutritional intake and physical activity are not adequately preserved [102]. The authors emphasized that GLP-1RAs should be accompanied by structured resistance training, adequate protein intake, and periodic assessment of muscle strength to mitigate potential declines in lean mass. Accordingly, when GLP-1RAs are used in populations at risk for muscle loss, general measures to help preserve skeletal muscle, such as maintaining habitual physical activity, incorporating resistance exercise training when feasible, and ensuring protein intake, may be beneficial. Resistance training may be especially synergistic with incretin-based weight-loss therapies. Recent data suggest that structured resistance exercise can attenuate reductions in lean mass during GLP-1–induced weight loss and may optimize improvements in body composition, highlighting the importance of integrating exercise-based muscle preservation strategies when prescribing these agents [103]. In older adults or individuals with low-activity levels, periodic assessment of muscle strength or physical function may help identify early declines, and interdisciplinary support (e.g., dietitians or rehabilitation specialists) can be considered based on individual needs [104].

There are concerns that older adults may be at risk for sarcopenic obesity, a condition characterized by excessive adiposity and low skeletal muscle mass [105]. The risk for sarcopenic obesity may be further exacerbated by weight cycling related to repeated treatment cessation, which may concomitantly exacerbate fat mass gains while reducing muscle mass. Maintaining muscle strength and medication guidance by comprehensive cardiac rehabilitation may be effective in older HFpEF patients receiving GLP-1RAs. Although outcome-based evidence is limited, routine imaging-based assessment of lean mass may also be recommended. Further studies are needed to clarify these points.

In HFpEF with obesity, moderate calorie restriction combined with high-quality nutrient intake and structured exercise training is recommended [27]. In the context of GLP-1RA therapy, appetite suppression can substantially reduce energy intake [106,107] (by approximately 16–39%), which may increase vulnerability to inadequate protein intake or malnutrition in older or low-activity patients. Regular nutritional assessment, monitoring of physical function, and early counseling are therefore advisable [108]. When GI-related adverse effects occur, short-term guidance, such as taking small, frequent, and easily digestible meals, may help maintain adequate intake [109].

Since direct evidence demonstrating a synergistic interaction between GLP-1RA therapy and CR remains limited, the combination therapy of GLP-1RAs and CR should, at present, be considered a strategy for hypothesis generation rather than an established evidence-based therapy.

Effective delivery of nutritional care requires coordinated multidisciplinary support. Within CR, physicians oversee the treatment plan, while nurses and physical therapists monitor clinical status, and dietitians provide specialized nutritional counseling and facilitate behavioral change [110]. Integrated care models combining cardiology assessment, medication optimization, dietitian-led nutrition therapy, and structured exercise support have demonstrated feasibility and improved adherence in outpatient HF management [111,112]. Collaboration with families, community resources, and allied health professionals further enhances the sustainability of lifestyle modification [113].

In sum, nutritional management should not be integrated—not isolated—within a comprehensive, team-based CR framework. Coupling dietary intervention with exercise therapy, pharmacotherapy, and ongoing functional monitoring is likely to enhance the overall quality of care in patients with HFpEF with obesity.

## 5. Potential Risks and Considerations

### 5.1. Adverse Effects: Gastrointestinal, Dehydration, and Hypoglycemia

Gastrointestinal disturbances represent the most prevalent adverse effects associated with GLP-1RAs, including upper GI symptoms, such as nausea and vomiting, and lower GI manifestations, including diarrhea and constipation. These events are observed in approximately 80% of individuals receiving GLP-1RA therapy across major weight loss randomized controlled trials (RCTs). In patients with T2DM, liraglutide, semaglutide, and tirzepatide are associated with significantly increased incidences of nausea, vomiting, and diarrhea [114]. The reported frequencies of nausea, diarrhea, and vomiting range from 15 to 30%, 10 to 15%, and 5 to 10%, respectively (Table 2) [115]. These effects appear to represent a class effect and exhibit dose dependency. They are generally transient, mild to moderate in intensity, and most commonly occur during treatment initiation or dose escalation [116,117].

Gastrointestinal adverse events constitute the leading cause of treatment discontinuation in clinical trials, with withdrawal rates reaching up to 12%. Real-world cohort studies demonstrate similar discontinuation frequencies, with nausea (35%) and constipation (29%) being the most frequently reported symptoms [118], indicating similar adverse effects between RCTs and clinical practices. In a retrospective cohort of 189 T2DM patients initiating subcutaneous semaglutide, 9.5% discontinued therapy due to GI intolerance, whereas adverse effects limited dose escalation in 5.8% [119]. Similarly, following a switch from another GLP-1RA to semaglutide, 10.4% of patients discontinued treatment because of GI-related adverse events [125]. Collectively, approximately 10% of patients discontinue semaglutide therapy as a result of GI-related adverse effects, a rate higher than that observed with other GLP-1RAs [120].

Among individuals with overweight or obesity, the frequency of GI-related adverse events appears to increase due to higher dosing regimens [126]. The pathophysiology underlying these events is multifactorial. Short-acting GLP-1RAs, primarily used in T2DM management, are associated with greater incidences of nausea and vomiting and lower rates of diarrhea compared with long-acting agents, which are administered for both T2DM and obesity. Nausea may arise from GLP-1 receptor activation within the central nervous system, whereas delayed gastric emptying and altered intestinal motility may further contribute to these manifestations [127]. However, a recent meta-analysis encompassing 55 RCTs with a total of 106,395 participants demonstrated that GLP-1RA therapy was associated with an increased risk of gastroesophageal reflux disease (GERD) (RR 2.19; 95% CI, 1.48–3.25) compared with placebo. Conversely, no significant associations with gastritis, esophagitis, GI ischemia, hemorrhage, ulceration, perforation, obstruction, paralytic ileus, or gastroparesis were identified [121].

Nausea, vomiting, and diarrhea can persist for several days, potentially leading to fluid depletion and electrolyte disturbances, thereby increasing the risk of dehydration. This dehydration may contribute to renal impairment, manifesting as either acute kidney injury or worsening of pre-existing chronic kidney disease [128]. Additionally, reduced food and fluid intake resulting from appetite suppression may further exacerbate dehydration [129]. Notably, liraglutide has been shown to exert a hypodipsic effect in rat models independent of its GLP-1–mediated impact on food intake [130]. This suppression of water intake is thought to occur partly through central nervous system GLP-1 receptor activation, consistent with findings from studies on food intake [131].

It is important to highlight several clinically relevant gastrointestinal adverse effects associated with GLP-1RAs. An association between GLP-1RAs and acute pancreatitis has been reported [132], and these agents should be discontinued if pancreatitis occurs. In addition, the U.S. Food and Drug Administration (FDA) has recently issued warnings regarding a potential risk of ileus with semaglutide and tirzepatide [133]. The risk of intestinal obstruction has also been reported to increase approximately 1.6 years after initiation of GLP-1RA therapy [134]. Gastroparesis is another recognized adverse effect and may persist even after discontinuation of treatment. Furthermore, cholelithiasis and cholecystitis are established adverse events associated with GLP-1RAs [135]. Because randomized controlled trials evaluating these agents are typically of relatively short duration, such adverse events may be underrecognized in clinical trial settings.

Although GLP-1RAs were initially developed as glucose-lowering agents for diabetes management, their primary mechanism in reducing blood glucose occurs through the stimulation of glucose-dependent insulin secretion. Importantly, inhibition of glucagon release by GLP-1RAs does not occur during hypoglycemic states [136], thereby explaining the reduced incidence of hypoglycemia compared with other antidiabetic classes. Clinical trials such as SUSTAIN-6 and PIONEER have demonstrated comparable rates of hypoglycemia between semaglutide-treated and placebo groups [9,61]. Notably, most hypoglycemic or severe hypoglycemic episodes were observed in patients concurrently receiving sulfonylureas or insulin or with concomitant use of each with other antidiabetic agents. Therefore, although GLP-1RAs alone carry a minimal risk of hypoglycemia, co-administration with sulfonylureas and/or insulin increases this risk. Dose adjustment of these agents before or during GLP-1RA titration is recommended to mitigate severe hypoglycemia [137]. A recent meta-analysis comparing GLP-1RAs with conventional antidiabetic drugs (including metformin, insulin, sulfonylureas, SGLT2 inhibitors, and DPP-4 inhibitors) identified tirzepatide, semaglutide, and liraglutide as the most potent agents for reducing HbA1c and fasting plasma glucose levels. Semaglutide was associated with a higher potential for hypoglycemia, whereas liraglutide and lixisenatide demonstrated a significantly lower hypoglycemia risk compared with traditional therapies, suggesting that liraglutide may represent the safer option for T2DM patients concerned about hypoglycemia [122].

Real-world data further support the low hypoglycemia risk associated with GLP-1RA therapy. In a Canadian observational cohort of 815 T2DM patients treated with semaglutide over six months, no increase in hypoglycemic events was observed [138]. Similarly, a retrospective study in Pennsylvania reported that both GLP-1RAs and SGLT2 inhibitors were associated with a lower risk of hypoglycemia than sulfonylureas and a risk comparable to that of DPP-4 inhibitors [139]. Even in patients with type 1 diabetes, adjunctive semaglutide improved glycemic control without elevating hypoglycemia risk compared with intensive insulin therapy alone [140]. Collectively, evidence from clinical trials, meta-analyses, and real-world studies indicates that GLP-1RAs have a favorable hypoglycemia profile; however, therapy should still be individualized based on patient-specific factors.

### 5.2. Use in Elderly and Frail Patients

Older adults often present with multiple comorbidities, functional and cognitive impairments, and age-related degenerative changes, all of which increase their susceptibility to adverse drug reactions and poor clinical outcomes. Most RCTs have not specifically focused on evaluating the effects of GLP-1RAs in elderly populations. In a network meta-analysis involving 41,654 patients with T2DM aged over 65 years, GLP-1RAs significantly reduced the risk of MACEs (RR 0.83; 95% CI 0.71–0.97) and body weight (mean difference [MD] −3.87 kg; 95% CI −5.54 to −2.21) compared to placebo [141]. Their efficacy in glycemic control in older adults was comparable to that in younger individuals; however, drug discontinuation due to adverse effects occurred more frequently among the elderly (OR = 0.67; 95% CI 0.47–0.96; *p* = 0.028) [101]. The increased incidence of GI intolerance was also observed in the SUSTAIN-6 trial [9]. Overall, the CV benefits and safety profiles of GLP-1RAs appear consistent across age groups, albeit with a slightly higher rate of adverse event-related discontinuation in older adults [142].

The potential influence of GLP-1RAs on depression has also been examined. In a recent study, GLP-1RA use was associated with a modestly lower risk of depression compared with DPP-4 inhibitors; however, no significant difference was found when compared with SGLT2 inhibitors [143]. Conversely, the EMPRISE study, which compared empagliflozin with cardioprotective GLP-1RAs, reported a greater absolute risk reduction in MI or stroke with empagliflozin, particularly among older patients [144]. Overall, GLP-1RAs remain a viable therapeutic option for older adults with T2DM, provided that patients are closely monitored for serious adverse events.

Frailty has emerged as a significant complication of diabetes in older individuals, who have a higher risk of frailty. Frailty represents a clinical state of diminished physiological reserve across multiple organ systems, increasing vulnerability to physical and metabolic stressors. Frailty is often characterized by unintentional weight loss, reduced muscle strength, and slower physical performance, and it is frequently accompanied by an elevated risk of hypoglycemia. According to the Fried criteria, frailty is diagnosed when at least three of the following are present: unintentional weight loss, exhaustion, low physical activity, weakness (measured by grip strength), and slowness (measured by gait speed) [93]. Three 1:1 propensity score-matched cohort studies using Medicare data compared SGLT2 inhibitors, DPP-4 inhibitors, and GLP-1RAs in older T2DM patients. Both SGLT2 inhibitors and GLP-1RAs demonstrated superior CV effectiveness compared to DPP-4 inhibitors across all frailty strata, with the greatest absolute benefit observed in the frailer patients. Although frailty was associated with higher rates of severe adverse events, these rates did not differ significantly between the three drug classes [145].

Frailty is also associated with sarcopenia, which extends beyond normal age-related muscle loss [146]. GLP-1RAs are associated with a significant reduction in body weight, fat mass, and lean mass. Among these agents, liraglutide was the only GLP-1RA to achieve significant weight loss without a significant decline in lean mass, whereas tirzepatide and semaglutide were most effective in reducing weight and fat mass but least effective in preserving lean mass [147]. Frail, obese older adults with T2DM may thus derive greater benefit from GLP-1RA therapy compared to malnourished individuals. Several guidelines provide nuanced recommendations for GLP-1RA use in this population. The International Diabetes Federation cautions against the use of GLP-1RAs in frail elderly patients, where further weight loss could be harmful [148]. Similarly, the Diabetes Canada guideline advises cautious use, noting that older adults treated with GLP-1RAs may be more susceptible to dehydration and fractures [123].

### 5.3. Long-Term Safety and Limitations of Current Evidence

Although GLP-1RAs are generally considered safe in individuals with obesity and/or T2DM, evidence from long-term studies remains inconsistent. In a meta-analysis by Li et al. involving 18,876 participants with T2DM, GLP-1RAs were associated with a significantly higher incidence of adverse events (hypoglycemia, nausea, vomiting, and diarrhea) over the study follow-up period. The risk of hypoglycemia was greater than that observed with placebo at 24–30 weeks (RR 1.61, 95% CI 1.36 to 1.91; *p* < 0.001, *I*^2^ = 68). Gastrointestinal symptoms such as nausea, vomiting, and diarrhea also occurred. Although seven cases of pancreatitis were reported, the incidence did not significantly differ between GLP-1RA and placebo groups [149]. Concerns have been raised regarding potential associations between GLP-1RA therapy and thyroid cancer; however, a multi-site cohort study involving more than 98,000 T2DM patients in Asia, Europe, and North America found no increased risk of thyroid cancer with GLP-1RA use during up to 3 years of follow-up [150]. In contrast, Gamborg et al. reported findings from a Danish cohort of 195,702 patients followed for up to 3.6 years, showing a higher incidence of certain cancers, including melanoma, colorectal, prostate, lung, and urinary tract neoplasms, among GLP-1RA users compared with those receiving DPP-4 inhibitors, particularly among women. Patients with GLP-1RA treatment had a higher risk of cancer (skin melanoma, colorectal, prostate, lungs, urinary system neoplasms) compared to patients treated with DPP-4 inhibitors, particularly in women. In a Danish nationwide cohort study by Gamborg et al., after 10 years of sustained therapy, the absolute risk of cancer was 25.5 (95% CI 23.3–27.4) per 100 patients treated with GLP-1 receptor agonists compared with 21.4 (95% CI 18.8–24.1) per 100 patients treated with DPP-4 inhibitors [124]. Although this difference suggested an association between GLP-1RA use and cancer incidence, the authors concluded that the finding was more likely attributable to improved survival among patients receiving GLP-1RAs rather than a carcinogenic effect of the treatment itself. Further studies in other ethnic groups and populations are needed to clarify the long-term safety profile of GLP-1RAs. Given that GLP-1RAs have only been studied for approximately two decades, the long-term safety and efficacy profiles of these agents remain to be fully established.

Despite the promising evidence from the GLP-1RA trials, particularly among patients with HFpEF and obesity or T2DM, the current evidence has some limitations. The observed benefits appear to be confined mainly to individuals with obesity, diabetes, and/or HFpEF, whereas studies involving patients with HFrEF have generally shown neutral outcomes. Moreover, therapeutic effects differ across various GLP-1RA agents, with newer agents such as semaglutide and tirzepatide exhibiting more favorable outcomes in HF populations. Most RCTs and CVOTs have relatively short follow-up durations, limiting conclusions regarding long-term efficacy and safety. Comparative studies between different GLP-1RAs or between GLP-1RAs and other HF therapies, aside from SGLT2 inhibitors, remain scarce. Consequently, GLP-1RA therapy in patients with HF should be tailored on an individual basis and guided by current clinical guidelines and consensus recommendations.

## 6. Future Directions

### 6.1. Research Questions on Synergy with Rehabilitation

The potential synergy between GLP-1RA treatment and rehabilitation should be explored further in future studies. Mechanistic studies should clarify how GLP-1RA–induced metabolic adaptations interact with exercise-mediated signaling pathways at molecular and cellular levels, the influence of GLP-1RA on exercise tolerance and muscle metabolism, and whether GLP-1RAs modify appetite and energy expenditure in HF patients who undergo CR. Clinical trials are needed to determine whether a combination of GLP-1RAs and rehabilitation improves exercise capacity, body composition, QOL, and the risk of mortality and morbidity beyond the effects of either intervention alone, particularly in HF patients. The safety of GLP-1RAs combined with rehabilitation should also be assessed, including the adverse events profile (hypoglycemia, dehydration, GI events).

Further studies in HF patients should focus on the effect of the combination of GLP-1RAs and CR on LV diastolic and systolic function, myocardial remodeling, and exercise tolerance. Whether the timing of GLP-1RAs initiation affects clinical outcomes in patients who go through HF rehabilitation programs should also be explored. In the context of rehabilitation programs, important gaps in the existing evidence include the impact of GLP-1RAs on muscle strength, mobility, and physical performance during rehabilitation in obese or diabetic patients as well as the effects of GLP-1RAs on body composition (muscle and fat mass) during the combination of GLP-1RAs and rehabilitation.

### 6.2. Need for Interventional Studies in HFpEF

Despite encouraging findings from recent clinical trials, there remains a critical need for large RCTs evaluating GLP-1RAs specifically for the treatment of HFpEF, with HF-related outcomes serving as primary endpoints. Most evidence for GLP-1RAs in CVD is derived from CVOTs in patients with T2DM or obesity [9,11,60,63,64,66,67,68], where heart-failure endpoints were secondary or exploratory. These trials were not designed to test hypotheses about HFpEF pathophysiology. Large RCTs with HF-specific primary endpoints are necessary to establish causality, quantify the magnitude of benefit (or harm), and provide the level of evidence required to change clinical practice guidelines and to inform regulatory and reimbursement decisions.

Mechanistically, GLP-1RAs may influence HFpEF progression through several biologically plausible pathways beyond weight reduction and glucose lowering, but these mechanisms remain incompletely validated in humans. Experimental and translational studies suggest GLP-1 and GLP-1 receptor signaling can improve endothelial function, reduce systemic and myocardial inflammation, attenuate cardiac fibrosis, enhance myocardial energetics and mitochondrial function, and promote natriuresis through renal actions [55,151,152]. Each of these effects targets core pathophysiologic features implicated in HFpEF—microvascular dysfunction, myocardial fibrosis, low-grade inflammation, and altered myocardial metabolism. Nevertheless, preclinical mechanistic data and post hoc clinical observations can only generate hypotheses; only adequately powered RCTs with prespecified mechanistic sub-studies (imaging, hemodynamic, biomarker panels, myocardial tissue sampling where feasible) can determine whether these pathways translate into clinically meaningful reductions in decompensation, functional decline, and mortality.

The rationale for conducting large RCTs specifically in patients with HFpEF without obesity or diabetes is equally strong. Existing studies showed inconsistencies among different GLP-1RAs and patient phenotypes. Benefits appear greater in obese or diabetic HFpEF patients than in lean and/or non-diabetic populations [153], suggesting a phenotype-specific response that requires confirmation in targeted RCTs. This raises the possibility that observed benefits are mediated predominantly by metabolic effects (weight loss, improved insulin sensitivity, or reductions in visceral adiposity) rather than direct myocardial or vascular effects. However, 20–40% of patients with HFpEF are lean or normoglycemic and may have pathophysiologic drivers (aging-related myocardial fibrosis, systemic microvascular rarefaction, hypertensive heart disease, or amyloid and infiltrative processes) that differ from those in metabolic HFpEF [154]. If GLP-1RAs exert cardioprotective actions that are independent of metabolic modulation, such as direct anti-inflammatory or anti-fibrotic effects, these ought to be demonstrable in non-obese/non-diabetic HFpEF cohorts. Conversely, if benefits are confined to metabolic phenotypes, exposing non-metabolic patients to potential side effects without benefit would be unethical. Therefore, phenotype-targeted RCTs are essential to delineate which HFpEF subgroups derive benefit, to avoid inappropriate extrapolation, and to enable precision medicine approaches.

To determine whether GLP-1RAs exert disease-modifying effects independently of weight loss, future HFpEF trials should include weight loss-matched control groups and enrollment of non-obese or weight-stable HFpEF cohorts.

Furthermore, safety, tolerability, and hemodynamic consequences may differ in lean or normoglycemic HFpEF patients. GLP-1RAs can cause GI adverse effects, volume depletion through reduced fluid intake, and changes in blood pressure—effects that may have different clinical implications in lean HFpEF patients who frequently have lower physiologic reserve. Long-term safety signals (renal function trajectories, arrhythmia risks, neurohormonal modulation) observed in diabetic populations may not generalize to non-diabetic HFpEF, necessitating dedicated safety monitoring and adjudication within trials powered to detect both efficacy and uncommon but clinically significant harms. Therefore, large-scale, phenotype-specific RCTs are crucial not only to establish efficacy but also to define optimal dosing, safety profiles, and mechanistic pathways in non-obese, non-diabetic HFpEF, ultimately guiding personalized therapy across the entire HFpEF spectrum. Even in the general HFpEF population, including those with obesity and T2DM, there are currently no large studies evaluating the long-term efficacy and safety of GLP-1RAs. Thus, specific interventional studies in the HFpEF population, with various phenotypes and long follow-up durations, are essentially needed.

### 6.3. Role in Personalized Medicine and Risk-Based Strategies

The identification of patients who can benefit from GLP-1RA treatment remains in the hands of the clinician. The risk-based strategies encompass careful patient selection, pre-treatment screening, appropriate dose initiation and titration, comprehensive patient education, and close monitoring for specific adverse events. Current guidelines recommend GLP-1RAs with proven CV benefit for individuals with T2DM who have ASCVD or CV risk factors, regardless of their HbA1c levels [73,74,75]. Given that GLP-1RAs are primarily glucose-lowering agents, the benefits of selecting these agents in T2DM patients should be weighed against those of other antidiabetic medicines. Although SGLT2 inhibitors are preferred in patients with HF or chronic kidney disease (CKD), GLP-1RAs may be more favorable in patients with higher ASCVD risk or obesity [153]. Caution is advised in severe CKD (eGFR  <  30 mL/min/1.73 m^2^) given the limited current data in this specific patient population [155]. In patients with CKD, liraglutide, semaglutide, and dulaglutide do not require dose adjustment in mild to moderate renal impairment. Risk-based algorithms may therefore be clinically useful in guiding therapy. For example, early initiation of GLP-1RA as first-line therapy is reasonable in a patient with T2DM, BMI > 30 kg/m^2^, and ASCVD. Conversely, among patients with modest obesity, low CV risk, and well-controlled glycemia, the selection of GLP-1RAs should consider the cost and the patient’s adherence.

The application of GLP-1RAs in HF requires careful phenotypic differentiation. The current evidence indicates that therapeutic response depends strongly on HF subtype, comorbidity burden, and body composition. In the guidelines, the use of GLP-1RAs is only recommended in HFpEF patients with obesity [75]. Specific studies on the HFpEF population in the future may help clinicians make decisions regarding HFpEF patients with or without obesity and diabetes. Meanwhile, current data for the use of GLP-1RAs in HFrEF patients are less favorable. Hence, clinical decision-making in the HF population should begin with detailed cardiac and metabolic phenotyping, including brain natriuretic peptide (BNP)/NT-proBNP measurement, echocardiographic classification, and obesity/diabetes status, to guide appropriate use. Risk-based strategies in HF thus emphasize selective use: GLP-1RAs may be considered in obese or diabetic HFpEF patients already on optimized guideline-directed therapy (including SGLT2 inhibitors), whereas their use in HFrEF should remain limited to clinical trial settings or under specialist supervision.

Initiation of therapy typically involves a low starting dose to reduce GI side effects, followed by gradual titration based on clinical response and tolerability [126]. Patients should be educated to take smaller, more frequent meals with adequate hydration. Dosing regimens vary between short-acting and long-acting formulations. Long-acting agents, such as dulaglutide and semaglutide, are generally preferred owing to their once-weekly dosing, which improves patient adherence and convenience [156]. Other potential side effects include injection site reactions, pancreatitis, gallbladder disease, such as cholelithiasis, and a theoretical increased risk for thyroid C-cell tumor; however, human data have not confirmed this risk [157]. Currently, the use of GLP-1RAs is contraindicated in individuals with a personal or family history of medullary thyroid carcinoma or multiple endocrine neoplasia syndrome type 2 [158]. Routine thyroid monitoring in patients taking GLP-1RAs is currently not recommended [159]. Clinicians should also be aware of patients with contraindications to GLP-1RAs (pregnancy, breastfeeding, pancreatitis, and severe renal impairment). Patients on GLP-1RAs may also need to hold the therapy before undergoing elective surgeries [160]. The use of GLP-1RAs may increase the risk of GI adverse events due to delayed gastric emptying. The holding duration will vary based on the frequency of GLP-1RA administration. Regular follow-up is essential to assess therapeutic response, monitor glycemic and CV outcomes, and manage adverse effects. Close clinical monitoring ensures the optimization of both efficacy and safety.

**Figure 4:**
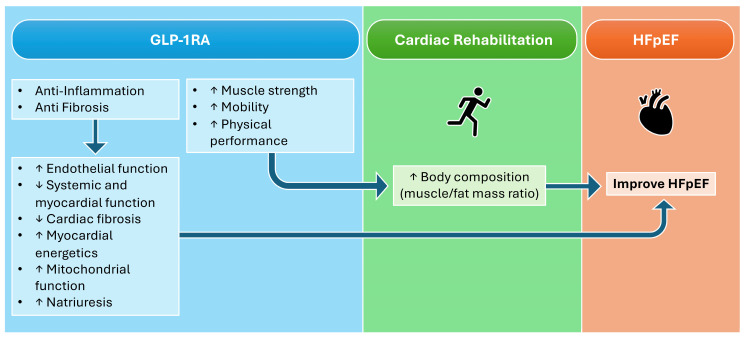
The benefit of GLP-1RAs combined with rehabilitation in HFpEF. GLP-1RA: glucagon-like peptide-1 receptor agonist; HFpEF: heart failure with preserved ejection fraction.

Beyond pharmacotherapy, the combination of GLP-1RAs with structured rehabilitation programs—encompassing aerobic and resistance exercise, dietary support, and behavioral counseling—may yield additive benefits in HF management. Recent mechanistic studies indicate that GLP-1RAs enhance exercise tolerance and muscle perfusion in HFpEF patients, potentially amplifying the gains achieved by CR [161]. This synergy arises from complementary physiological mechanisms: GLP-1RAs improve metabolic efficiency and reduce adipose-driven inflammation, while exercise training enhances endothelial function and skeletal muscle oxidative capacity. An integrated approach combining both therapies could improve QOL, cardiorespiratory fitness, and long-term outcomes in HFpEF, aligning with the principles of precision CV medicine (Figure 4). Personalized and risk-based strategies are essential for optimizing GLP-1RA therapy across the clinical spectrum. Future directions should focus on biomarker- and genotype-guided algorithms to identify ideal responders and on interventional trials combining pharmacologic and rehabilitative approaches to enhance long-term cardiometabolic health.

### 6.4. Limitations

Several limitations of the current evidence should be acknowledged. First, most randomized clinical trials evaluating GLP-1RAs in heart failure have focused primarily on obese patients with HFpEF, and their applicability to non-obese HFpEF populations remains uncertain. Second, evidence supporting the use of GLP-1RAs in HFrEF is limited and largely derived from small, randomized trials and observational studies. Third, although improvements in symptoms, exercise capacity, and body composition have been demonstrated, robust data on long-term mortality and heart failure hospitalization outcomes remain insufficient. Finally, the proposed integration of GLP-1RA therapy with cardiac rehabilitation and nutritional strategies is supported primarily by mechanistic rationale and indirect clinical evidence and therefore requires confirmation in prospective randomized studies.

## 7. Conclusions

Heart failure, particularly HFpEF, remains a major unmet clinical challenge, driven by complex systemic abnormalities, including metabolic dysfunction, inflammation, endothelial impairment, and skeletal muscle dysfunction. GLP-1RAs, originally developed for T2DM, have emerged as pleiotropic agents with favorable effects on body weight, inflammation, vascular function, and metabolic efficiency.

Accumulating evidence suggests that GLP-1RAs may provide clinically meaningful benefits in selected HF populations, especially patients with HFpEF and obesity. These benefits appear to be mediated predominantly through indirect systemic mechanisms rather than direct myocardial actions, targeting key processes that contribute to exercise intolerance and functional limitation. The integration of GLP-1RAs with CR therefore may represent a promising, synergistic strategy to enhance functional recovery and quality of life.

However, the use of GLP-1RAs in HF should be individualized, as evidence in HFrEF remains limited. Future phenotype-specific randomized trials and mechanistic studies are needed to define their optimal role within precision-based HF rehabilitation strategies. While data from randomized clinical trials for obese patients with HFpEF are evidence-based, the combination therapy of GLP-1RAs and CR is a strategy for hypothesis generation. Further studies are needed to clarify this point.

Importantly, this review emphasizes the potential synergy between GLP-1RA therapy and cardiac rehabilitation, including structured exercise training and nutritional support aimed at preserving skeletal muscle mass and physical function. Such multidisciplinary integration may be particularly relevant in older patients with obesity-related HFpEF, in whom prevention of sarcopenia and maintenance of functional capacity represent key therapeutic goals. This integrated pharmacological, rehabilitation, and nutritional approach supports a phenotype-specific strategy for HFpEF management and represents a key distinguishing perspective of the present review.

## Figures and Tables

**Table 1:** A comparative table of recommendations for GLP1RA use based on various guidelines.

Guidelines	Recommendations	Class, Level of Evidence (LOE)
The ADA Standards of Care in Diabetes 2026 [70]	T2DM with established ASCVD or CKD GLP-1RAs with demonstrated cardiovascular disease benefit For CV risk reduction and/or as glucose-lowering treatment	LOE A
The 2023 ESC Guideline for The Management of CVD In Patients with Diabetes [71]	T2DM with ASCVD	I, A
	GLP-1RAs with proven CV benefit	
	For CV event reduction	
	Independent of baseline or target HbA1c and concomitant glucose-lowering medication	
	T2DM, without ASCVD, with a calculated 10-year CVD risk ≥10%	IIb, C
	GLP-1RAs (not specified)	
	For CV risk reduction	
	T2DM at risk of or with HF	IIa, A
	Lixisenatide, liraglutide, semaglutide, exenatide ER, dulaglutide, efpeglenatide	
	As glucose-lowering treatment	
JCS/JHFS 2025 Guideline on Diagnosis and Treatment of Heart Failure [72]	HF (LVEF ≥ 45%) and obesity	IIa, B-R
	Semaglutide	
	To reduce CV death and HF rehospitalization	
	HF (LVEF ≥ 50%) and obesity	IIa, B-R
	Tirzepatide	
	To reduce CV death and HF rehospitalization.	
CCS/CHFS 2025 Guideline Update for Pharmacologic Management of Heart Failure With Nonreduced Ejection Fraction (LVEF > 40%) [73]	Symptomatic HF, LVEF ≥ 45%, body mass index ≥ 30 kg/m^2^ Evidence-based GLP-1RAs with proven efficacy To reduce the risk of HF hospitalization and to improve QoL	Weak recommendation; moderate-quality evidence

Class IIa: There is a high probability of efficacy/usefulness based on evidence and opinion. Class IIb: usefulness/efficacy is less well established by evidence/opinion. B-R: Demonstrated by one or more randomized clinical trials. ADA: American Diabetes Association; T2DM: type 2 diabetes mellitus; ASCVD: atherosclerotic cardiovascular disease; CKD: chronic kidney disease; GLP-1RA: glucagon-like peptide-1 receptor agonists; HF: heart failure; CV: cardiovascular; HbA1C: hemoglobin A1C; LOE: level of evidence; HFpEF: heart failure with preserved ejection fraction; HFrEF: heart failure with reduced ejection fraction; ESC: European Society of Cardiology; JCS/JHFS: Japanese Circulation Society/Japanese Heart Failure Society; LVEF: left ventricular ejection fraction; CCS/CHFS: Canadian Cardiovascular Society/Canadian Heart Failure Society; QOL: quality of life.

**Table 2:** Adverse events and special precautions of GLP-1RA therapy.

Author, Year [Ref]	Study	Adverse Effects	Frequency/HR
The most frequent adverse effects: Gastrointestinal tract			
Bettge et al. 2017 [115]	Systematic analysis, GLP-1RAs	Nausea	15–30%
		Diarrhea	10–15%
		Vomiting	5–10%
Sorensen et al. 2025 [118]	Real world cohort, GLP-1RAs	Nausea	35%
		Constipation	29%
Williams et al. 2021 [119]	Retrospective cohort, semaglutide	Discontinued due to gastrointestinal intolerance	9.5%
Smits et al. 2021 [120]	Systematic review, semaglutide	Discontinued due to gastrointestinal adverse effect	10%
Chiang et al. 2025 [121]	Meta-analysis, GLP-1RAs	Gastroesophageal reflux disease	HR 2.19 vs. placebo
Rarer and less frequent adverse effects			
Ren et al. 2025 [122]	Meta-analysis, GLP-1RAs	Hypoglycemia	Semaglutide > liraglutide or exenatide
Ivers et al. 2018 [123]	Diabetes Canada 2018 Clinical Practice Guideline	Susceptible to dehydration and fracture	In frailty elderly
Gamborg et al. 2025 [124]	Danish cohort, GLP-1RAs	Higher incidence of certain cancer: melanoma, colorectal, prostate, lung, and urinary tract neoplasms	GLP-1RAs > DPP-4 inhibitors, particularly in women

HR: hazard ratio; GLP-1RA: glucagon-like peptide 1 receptor agonist; DPP-4: dipeptidyl peptidase IV.

## Data Availability

No new data were created or analyzed in this study. Data sharing is not applicable to this article.

## Abbreviations

The following abbreviations are used in this manuscript:

ASCVD	Atherosclerotic cardiovascular disease
BMI	Body mass index
BNP	Brain natriuretic peptide
cAMP	Cyclic adenosine monophosphate
CI	Confidence interval
CKD	Chronic kidney disease
CR	Cardiac rehabilitation
CV	Cardiovascular
CVD	Cardiovascular disease
CVOTs	Cardiovascular outcome trials
DM	Diabetes mellitus
DPP-4	Dipeptidyl peptidase-4
eGFR	Estimated glomerular filtration rate
ET-1	Endothelin-1
GI	Gastrointestinal
GLP-1	Glucagon-like peptide-1
GLP-1R	Glucagon-like peptide-1 receptor
GLP-1RAs	Glucagon-like peptide-1 receptor agonists
HF	Heart failure
HFpEF	Heart failure with preserved ejection fraction
HFrEF	Heart failure with reduced ejection fraction
HR	Hazard ratio
KCCQ	Kansas City Cardiomyopathy Questionnaire
LV	Left ventricle
LVEF	Left ventricular ejection fraction
MACE	Major cardiovascular events
MAPK	Mitogen-activated protein kinase
MI	Myocardial infarction
NO	Nitric oxide
NT pro-BNP	*N*-terminal pro-brain natriuretic peptide
PI3K	Phosphatidylinositol-3-kinase
PKA	Protein kinase A
QOL	Quality of life
RCTs	Randomized controlled trials
RR	Relative risk
SGLT2	Sodium glucose transporter-2
T2DM	Type 2 diabetes mellitus
VO_2_	Volume of oxygen
